# Segzoo: a turnkey system that summarizes genome annotations

**DOI:** 10.1093/bioinformatics/btag207

**Published:** 2026-05-05

**Authors:** Mickaël Mendez, Yushan Liu, Marc Asenjo Ponce de León, Michael M Hoffman

**Affiliations:** Department of Computer Science, University of Toronto, Toronto, ON M5S 3G4, Canada; Princess Margaret Cancer Centre, University Health Network, Toronto, ON M5G 1L7, Canada; Princess Margaret Cancer Centre, University Health Network, Toronto, ON M5G 1L7, Canada; Department of Mathematics, University of Toronto, Toronto, ON M5S 2E4, Canada; Department of Statistical Sciences, University of Toronto, Toronto, ON M5G 1Z5, Canada; Princess Margaret Cancer Centre, University Health Network, Toronto, ON M5G 1L7, Canada; Facultat d’Informática de Barcelona, Universitat Politècnica de Catalunya, Barcelona, 08034, Spain; Department of Computer Science, University of Toronto, Toronto, ON M5S 3G4, Canada; Princess Margaret Cancer Centre, University Health Network, Toronto, ON M5G 1L7, Canada; Department of Medical Biophysics, University of Toronto, Toronto, ON M5G 1L7, Canada; Vector Institute, Toronto, ON M5G 0C6, Canada

## Abstract

**Motivation:**

Segmentation and automated genome annotation (SAGA) techniques, such as Segway and ChromHMM, assign labels to every part of the genome, identifying similar patterns across multiple genomic input signals. Inferring biological meaning in these patterns remains challenging. Doing so requires a time-consuming process of manually downloading reference data, running multiple analysis methods, and interpreting many individual results.

**Results:**

To simplify these tasks, we developed the turnkey system Segzoo. As input, Segzoo only requires a genome annotation file in browser extensible data (BED) format. It automatically downloads the rest of the data required for comparisons. Segzoo performs analyses using these data and summarizes results in a single visualization.

**Availability and implementation:**

The source code for Python ≥ 3.7 on Linux is freely available for download at https://github.com/hoffmangroup/segzoo under the GNU General Public License (GPL) version 2. Segzoo is also available in the Bioconda package segzoo: https://anaconda.org/bioconda/segzoo. We have deposited in Zenodo the version of the Segzoo source which produced the results in this article (https://doi.org/10.5281/zenodo.10988775), other code and data used to produce the results (https://doi.org/10.5281/zenodo.10477083), and the results (https://doi.org/10.5281/zenodo.10477106).

## 1 Introduction

Segmentation and automated genome annotation (SAGA) methods (Libbrecht et al. 2021), such as Segway (Hoffman et al. 2012) and ChromHMM (Ernst and Kellis 2012), partition the genome into segmentation made of non-overlapping segments. The methods assign a label to each segment such that the segments sharing the same label exhibit similarities in the input data. As the input data can consist of complex combinations of chromatin immunoprecipitation sequencing (ChIP-seq) data (Barski et al. 2007, Johnson et al. 2007, Robertson et al. 2007) or RNA sequencing (RNA-seq) data, inferring a biological role for a label requires comparing the segmentation against multiple reference datasets. This requires a non-trivial amount of toil (Rau 2016), including downloading reference data. It also involves orchestrating the use of these data with a variety tools, such as Segtools (Buske et al. 2011) and BEDTools (Quinlan and Hall 2010), potentially through the development of custom scripts. Interpretation requires simultaneous consideration of multiple visualizations and analyses.

To help assign biological hypotheses to SAGA labels, we designed the turnkey system Segzoo. Segzoo requires only a genome segmentation file as input to generate all the visualizations. Segzoo uses Go Get Data (GGD; Cormier et al. 2021) to automatically download all required data for these analyses and produces an easy to interpret figure which reveals patterns of segmented regions. Here, we demonstrate the use of Segzoo to quickly identify promoter features from a Segway annotation of the H1 human embryonic stem cell line.

## 2 Methods

Segzoo summarizes genome annotation through a four-step workflow. First, it uses GGD to download reference data for the genome assembly of the segmentation. Second, Segzoo generates segmentation-centric summary statistics using Segtools and BEDTools (Quinlan and Hall 2010). Third, Segzoo parses the output of each tool and summarizes them into one table. Fourth, Segzoo plots the table as a compact figure to allow rapid biological hypothesis assignment to SAGA labels.

### 2.1 Dependencies

In addition to a segmentation, Segzoo needs a gene annotation and a reference genome to perform analyses. Segzoo uses GGD to automatically download the data dependencies and Conda (Conda contributors 2022; https://docs.conda.io/projects/conda/) to automatically install the software dependencies. Segzoo uses the Snakemake (Köster and Rahmann 2012) workflow manager to automatically perform the necessary steps. Using Snakemake also allows taking advantage of multiprocessing to accelerate the analyses.

### 2.2 Analysis tools

Segzoo uses Segtools to perform a series of analyses. It combines the results of segtools-aggregation, segtools-gmtk-parameters, segtools-overlap, and segtools-length-distribution into a single plot. Segzoo also uses BEDTools’s (Quinlan and Hall 2010) bedtools nuc command to calculate the nucleotide frequency and G + C content for each segmentation label.

### 2.3 Input data

Here, we demonstrate the use of Segzoo by creating a Segway annotation of the H1 human embryonic stem cell line. To do this, we used ENCODE ChIP-seq data for CTCF (ENCFF332TNJ), H3K27me3 (ENCFF417VQQ), H3K4me1 (ENCFF584AVI), H3K27ac (ENCFF919FBG), and H3K4me3 (ENCFF760NUN). We trained the 10-label Segway model with the following parameters: segway--num-instances = 10 --resolution = 10 --minibatch-fraction = 0.01 --num-labels = 10. We set the environment variable SEGWAY_RAND_SEED = 22426492 to make the results reproducible.

**Figure 1:**
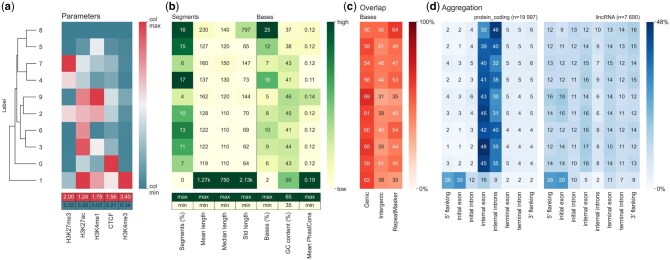
Segzoo summary of Segway H1 human embryonic stem cell line annotation. Numeric values in each heatmap represent various summary statistics for a label. (a) *Top*: Gaussian mean parameters from Segway trained model. Column-normalized values, with minimum and maximum values mapped to the range 0 to 100. *Left*: Dendrogram indicating hierarchical clustering on rows from weighted pair group method with arithmetic average (WPGMA; Sokal and Michener 1958). *Bottom*: Maximum and minimum values of each column. (b) *Top*: Heatmap of statistics summarizing segments. Grouped by segment: fraction of segments annotated with the label (“segments”), mean segment length (“mean length”), median segment length (“median length”), standard deviation of segment length (“std length”). Grouped by base: fraction of bases annotated with the label (“base pairs”), G + C content, mean PhastCons (Siepel et al. 2005) conservation score (“mean PhastCons”). The letter “k” indicates thousands. *Bottom*: Indication of maximum and minimum values of the color scale for each column from one of: column maximum value (“max”), column minimum value (“min”), other value (a number). (c) Fractional overlap of each label with genic, intergenic, and RepeatMasker (Smit et al. 2010; https://www.repeatmasker.org/) regions. (d) Fractional overlap between segments and components and flanking regions of GENCODE (Harrow et al. 2012) protein-coding genes (left) and long intergenic non-coding RNA (lincRNA) genes (right). Row-normalized values per table, with values summing up to 100.

We used Segzoo to generate a detailed summary figure (Fig. 1) with the following single command:

segzoo --parameterstraindir/params/params.params \

--dendrogramannotdir/segway.bed.gz

To plot the Parameters heatmap (Fig. 1a), we used the --parameter option with Segway’s trained parameter file, params.params. The file contains the mean of the Gaussian distribution learned by Segway for each pair of dataset and label. The other heatmaps (Fig. 1b–d) used the output of segway annotate, segway.bed.gz.

## 3 Results

The Segzoo visualization consists of an array of heatmaps, with rows representing SAGA labels and columns representing different quantitative descriptions of those labels (Fig. 1). The first heatmap, “Parameters”, indicates the mean parameters of the Gaussian distributions learned for each label on each input dataset (Fig. 1a). The other heatmaps summarize other aspects of the output segmentation, including summary statistics of the segments (Fig. 1b), proportion of bases in genic regions (Fig. 1c), and overlap between segments and components of GENCODE genes—both protein-coding genes (Fig. 1d, left), and lincRNAs (Fig. 1d, right). To simplify the interpretation of labels with similar patterns, Segzoo automatically performs row-wise and column-wise hierarchical clustering on the Parameters table. All the heatmaps share the same row order to simplify the interpretation of all the descriptions for a given label.

Here, Segzoo allows us to quickly see that label 1 has several promoter features (Fig. 1). First, label 1 has the highest mean H3K4me3 signal (Fig. 1a). This signal often indicates promoter regions (Liang et al. 2004). Second, label 1 has the highest G + C content (Fig. 1b), another feature of promoter regions (Akan and Deloukas 2008). Label 1 has the highest mean length and median length. We don’t expect this for a promoter region. Label 1, however, has the highest length standard deviation and the fewest segments. This suggests that Segway annotated not only promoter regions, but also long genomic regions with the label 1. Third, the Overlap table shows the segments with the label 1 mostly annotate genic regions (Fig. 1c). Fourth, the Aggregation table shows an enrichment of label 1 at the 5*'* end of both protein coding genes and lincRNAs (Fig. 1d). The four heatmaps together help generate the hypothesis that label 1 annotates promoter regions. Interestingly, we can also see easily that label 9 has promoter features such as high H3K4me3 signal, high G + C content, and frequent overlap at lincRNA 5*'* ends.

## 4 Conclusion

Segzoo simplifies the process of assigning a biological hypothesis to SAGA labels. To simplify the process, it generates a compact visualization that summarizes the overlap between a SAGA annotation and multiple reference datasets. Segzoo automatically download the reference datasets via standardized recipes with GGD, which makes it easier to attain higher degrees of reproducibility. For example, this work meets the silver standard of reproducibility (Heil et al. 2021).
